# Resting-state functional connectivity of the dorsal frontal cortex predicts subcortical vascular cognition impairment

**DOI:** 10.18632/oncotarget.21855

**Published:** 2017-10-16

**Authors:** Xiaopeng Hu, Xia Zhou, Chao Zhang, Haibao Wang, Yongqiang Yu, Zhongwu Sun

**Affiliations:** ^1^ Department of Radiology, The First Affiliated Hospital of Anhui Medical University, Anhui, China; ^2^ Department of Neurology, The First Affiliated Hospital of Anhui Medical University, Anhui, China

**Keywords:** multivariate pattern analysis, dorsal frontal cortex, vascular cognition impairment, subcortical

## Abstract

Functional magnetic resonance imaging (fMRI) studies have revealed group differences in the frontal area between the subcortical vascular cognition impairment (SVCI) patients and the controls. However, most of the existing research focused on average differences between the two groups, and therefore had limited clinical applicability. The aim of our study was to investigate whether inter-regions functional connectivity of the dorsal frontal cortex (DFC) can be used to discriminate the SVCI from the controls at the level of the individual. Thirty-two SVCI patients and 32 demographically similar healthy individuals underwent resting-state functional magnetic resonance imaging. The DFC, derived from a prior atlas, was divided into 10 clusters. Features based on DFC were obtained through functional connectivity analysis between pairs of DFC. A nonlinear kernel support vector machine was used for classification and validated using 8-fold cross validation. An excellent classification accuracy was obtained from both the left and the right DFC functional connectivity (accuracy=75.07%, sensitivity=81.57% and specificity=61.71%; accuracy=45.38%, sensitivity=60.74% and specificity=39.48%; P<0.001). These findings shed further light on the pathogenesis of SVCI and showed promising classification performance using machine learning analysis based on DFC fMRI data, which may be useful for the differentiation of SVCI.

## INTRODUCTION

Subcortical vascular cognition impairment (SVCI) is characterized by executive dysfunction, which was consistently thought to be associated with the dysregulation in frontal-subcortical loop [1]. In these loops, the frontal cortex exerts key high-level top-down control by influencing processing in other brain regions and is also considered to be the main driving force of the subcortical region [2]. At the neuroanatomy level, the frontal cortex consists of the dorsal frontal cortex (DFC) and the ventral frontal cortex (VFC), which play different roles in cognition and behavior. The VFC is considered to be mainly involved in valuation processes and language [3], while the DFC is regarded as an important brain structure in attention processing and cognition controls, both of which are significantly disrupted in patients with SVCI [4]. Several lines of evidence have showed the abnormal structural as well as functional impairment in the area of the DFC in SVCI patients, underlining its essential roles in the pathogenesis of SVCI [5–7]. However, these studies did not allow for direct assessment of specific DFC region to SVCI. According to previous study, the DFC contains several spatially separated regions in each hemisphere in both humans and macaques, based on diffusion-weighted magnetic resonance imaging (DW-MRI) and functional MRI (fMRI) techniques [8].

Functional MRI, especially the resting-state fMRI provides us a promising viewpoint to explore the function alteration of SVCI. However, in our daily life, the SVCI patients are prone to be ignored, especially during the early stages, due to the subtle clinic symptom or obscure onset. Therefore, it is necessary to find an objective biomarker, which could be used to assist the diagnosis of SVCI and to improve the accuracy. Multivariate pattern analysis (MVPA) is a promising and potentially powerful data-driven tool in clinical research that permits the differentiation of patients from healthy controls at individual subject level [9]. The most common pattern recognition used MVPA in neuroimaging literature is support vector machine (SVM), which has been successfully applied to classify various neuropsychiatric disorders and achieved good classification accuracy [10]. For resting-state functional MRI, functional connectivity, measured by the correlation of two functional MRI time series, has been used for the discrimination of several neurological and psychiatric disorders, including Alzheimer's disease, epilepsy and depression [11–13]. However, to the best of our knowledge, there is no study for automatic identification of the SVCI and healthy controls.

Thus, in the present study, we aim to examine DFC functional connectivity maps in differentiating SVCI patients from healthy controls and to find out which side of the DFC brain functional connectivity contribute more to the discrimination. We also sought to examine the relationship between identified group differences in DFC region and behavioral measures of cognition.

## RESULTS

### Demographic and clinical characteristics

Demographic and clinical characteristics for all of participants are shown in Table 1. No significant differences were found in age, gender ratio and education years between the SVCI patients and healthy controls (p>0.05). Compared to healthy controls, SVCI patients present significantly lower Cambridge Cognitive Examination-Chinese version (CAMCOG-C), Mini-Mental State Examination (MMSE), Clinical Dementia Rating (CDR) scores and higher Activities of Daily Living (ADL) scores, all of which indicated the impaired global cognition. Additionally, in the subtypes of CAMCOG-C, SVCI patients also exhibited lower scores in praxis item compared to the controls, which suggested the deficit in executive function of SVCI patients.

**Table 1:** Demographic and clinical characteristic of SVCI patients and healthy controls

Variables		SVCI (n=32)	Controls (n=32)	p-Value
Age (Years)		70.09±8.26	68.87±7.05	0.557^b^
Gender (F/M)		18/14	14/18	0.454^a^
Years of education		8.47±3.16	10.09±2.98	0.670^b^
CAMCOG-C		76.78±9.26	92.83±4.63	0.002^b^
	praxis	8.75±2.37	11.28±0.92	<0.001^b^
MMSE		23.78±2.66	28.38±1.10	<0.001^b^
ADL		25.47±7.42	20.19±0.59	<0.001^b^
CDR		0.5(0.5-2.0)	0	<0.001^c^

MMSE=Mini-Mental State Examination; CAMCOG-C=Cambridge Cognitive Examination-Chinese version. ADL=Activities of Daily Living scale.

GDS=Global Deterioration Scale; CDR=Clinical Dementia Rating.

^a^ two-tailed Pearson chi-square test, ^b^ two-sample two-tailed t-test, ^c^ Mann-Whitney U test.

### Classification results

**Figure 1:**
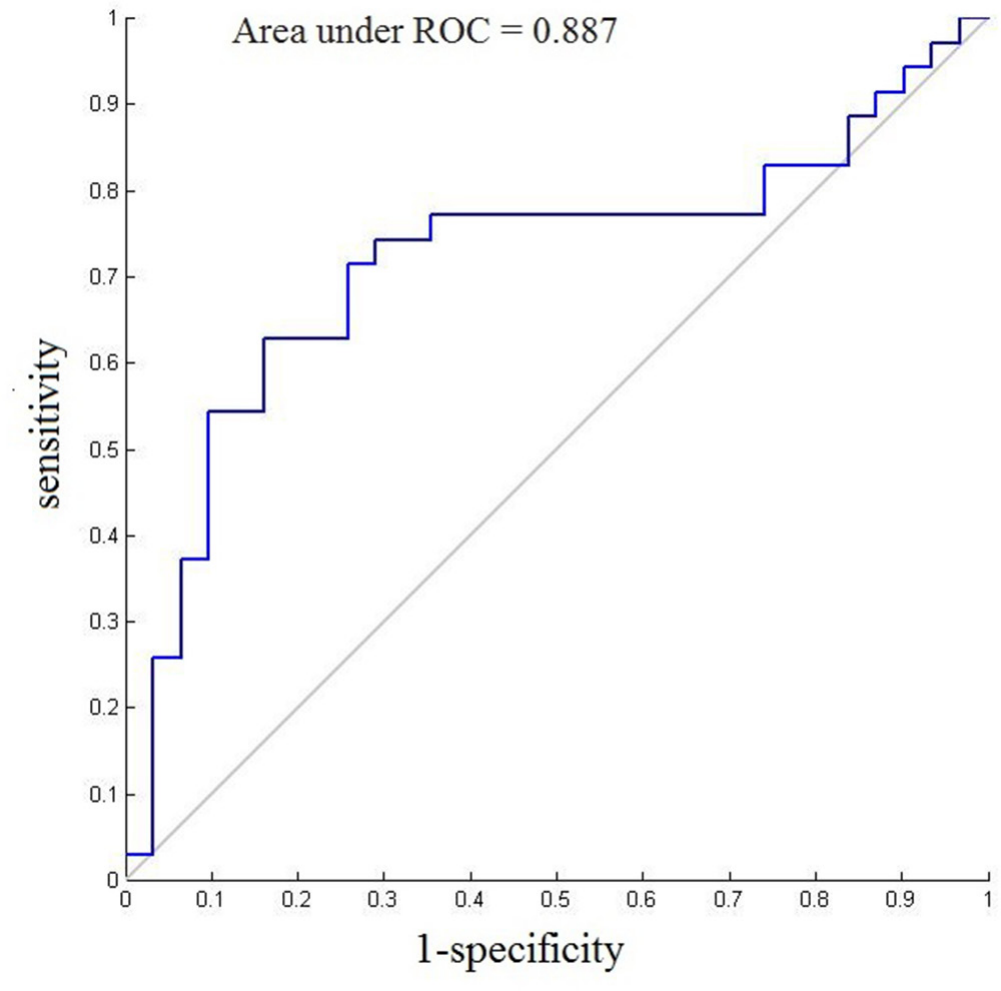
ROC curves and AUC of the final SVM model (the left DFC) for each patient

The classification results indicated that the final correct classification rate of the training data set was 45.38% using the right DFC discriminating functional connections, while the classification rate in the left DFC was up to 75.07% (Table 2). Receiver operating characteristic (ROC) showed excellent sensitivity and specificity when classifying connectivity features between groups (60.74% sensitivity and 39.48% specificity for the right DFC, p<0.001; 81.57% sensitivity and 61.71% specificity for the left DFC, p<0.001). We also examined the correct classification rate using both the left and the right DFC. The accuracy was 58.46% with the usage of the Regions of interest (ROIs) of both sides, which was intermediate between the right DFC and the left DFC. The area under ROC curves (AUCs) of the classifier for the right DFC and the left DFC were 0.814 and 0.887, respectively. The ROC curve for the left classifier is shown in Figure 1.

**Table 2:** Classification results in 8-fold cross validation using the functional connectivity maps of the DFC

Features	Accuracy (%)	Sensitivity (%)	Specificity (%)	AUC
the left DFC	75.07	81.57	61.71	0.887
the right DFC	45.38	60.74	39.48	0.814
the left D+the right DFC	58.46	71.43	48.39	0.877

### Voxel-based morphometry results

Voxel-based morphometry (VBM) results are shown in Table 3. No significant difference was found in the mean gray matter volume of both sides of DFC between the SVCI and the controls using the two sample *t* test.

**Table 3:** VBM analysis showing the difference of the subregions of DFC volume between SVCI patients and the controls

ROIs	Subregions of DFC	p	t
ROI 1	SMA	0.35647	-0.92883
ROI 2	pre-SMA	0.4244	-0.80396
ROI 3	area 9	0.43384	-0.7876
ROI 4	area 10	0.88732	-0.14226
ROI 5	area 9/46d	0.66624	-0.43332
ROI 6	area 9/46v	0.46504	-0.73497
ROI 7	area 46	0.45793	-0.74678
ROI 8	area 8d	0.77892	-0.28192
ROI 9	rostral PMd	0.47772	-0.71417
ROI 10	area 8v	0.66459	-0.43561

### Correlation analysis result

**Figure 2:**
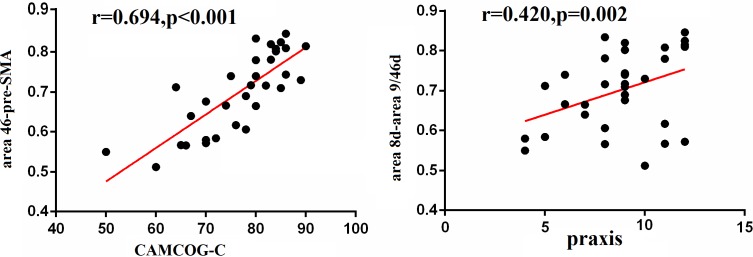
Correlation between cognition and functional connectivity in the DFC Pearson correlation analyses revealed that CAMCOG-C scores positively correlated with the functional connectivity between area 46 and pre-SMA, and the praxis function positively correlated with the functional connectivity between area 8d and area 9/46d.

There was a positive correlation between CAMCOG-C and the strength of functional connectivity (area 46-pre-SMA) in the left DFC (r=0.694, p<0.0001). We also found a trend for a positive relationship between praxis function and the connectivity between area 8d and area 9/46d (r= 0.420, p=0.002) (Figure 2).

## DISCUSSION

To the best of our knowledge, the current study is the first to examine the capability of SVM with functional connectivity in bilateral DFC in distinguishing patients with SVCI from healthy subjects. By identifying the subregional differences in both the left and the right hemispheres of brain DFC functional connectivity patterns, the present study demonstrates that MVPA allows discrimination between SVCI patients and healthy controls at a relatively high level of accuracy.

MVPA is an increasingly popular analytic technique in neuroimaging field, which renders a high discriminative power by considering spatially distributed patterns of brain activity instead of focusing on an isolated voxel. In the current study, we used MVPA based on fMRI and achieved 75.07% and 45.38% prediction accuracy in the left and the right DFC, respectively, for identifying SVCI patients. The excellent classification accuracy and sensitivity may imply the essential roles of the DFC, especially the left DFC in identifying the SVCI and also indicate the critical roles in the pathophysiology of SVCI. The DFC consists of 10 subregions connected to each other, which may be seen as the modularity. Integration within subregions allows faster local processing, while sparse connections between subregions reduce the efficiency. It is thus suspected that a less modular may decrease the development of a functional brain network and directly result in cognitive impairments.

To further explore whether the brain function alteration in DFC in SVCI patients was induced by structural atrophy, the gray matter in DFC was examined. No significant difference was found between the SVCI and the controls. Taking both the function and structure into consideration, we suggested that the aberrant function in DFC may contribute more to the pathophysiology of SVCI. The high capability of SVM methods in combination with functional connectivity metrics in our study may also indicate the important roles of DFC on SVCI. Furthermore, it is worth noting that the accuracy of the left DFC was greater than that of the right DFC. It is acceptable that there is a hemispheric difference in the human brain function. Compared to the right DFC, the left DFC may play leading roles in executive function, which has been shown by Stroop test [14]. Apparently, our results of the DFC classifier were consistent with those of a previous study [14], indicated the left DFC as the dominant hemisphere in execution. Thus, the dysfunction of the left DFC may lead to the executive impairment, which is the hallmark symptom of SVCI.

We also found that strength of functional connectivity between area 46 and pre-SMA was correlated with clinical measures. Area 46 is a hub region located in the anterior middle frontal gyrus, which has been suggested to possess extensive intracortical as well as fronto-subcortical connections [15]. The correlation between the functional connectivity metrics and the clinical measures supports its central hub role on the global cognition of SVCI, with flexible integration and projection of information. Moreover, in the DFC the connectivity between the area 8d and area 9/46d was also correlated with the executive function. Area 8d was located in the superior frontal gyrus. Clinical research has shown that patients with lesions in the superior frontal gyrus had worse executive function [16]. Our findings agree with those of previous studies, suggesting that the altered functional connectivity may be the reason for executive function impairments. Taken together, these results and our previous findings provide evidence that the DFC may consider being the key characteristics region in SVCI patients, which was attributable to the high discriminative accuracy of its functional connectivity based on MVPA.

The classification results of this study using MVPA based on DFC resting-state functional connectivity are encouraging. However, there are still some limitations needed to be clarified, such as scanner variability, micro-head movement and some physiological noises and so on. Although we have undertaken a set of proven strategies, such as nuisance regressors at the first level, noting the patients to be quite repeatedly, to counteract the effect of group differences in movement, it is still impossible to achieve an ideal state. Furthermore, considered the limited sample and the known groups of subjects, the supervised classification methods were used to analyze the data, which was prone to inter-user bias, while the unsupervised classification can perform discrimination without any prior labeling knowledge, so future study with a larger sample size using the unsupervised classification combined with the supervised classification would yield more powerful results. In addition, because of the small sample (64 subjects in total) in the study, the obtained classifier was only specific to the current data and not general enough. Future investigations with large database and the integration of functional connectivity with other neuroimaging methods will be needed to confirm these preliminary findings.

## MATERIALS AND METHODS

### Participants

Thirty-two right-handed SVCI patients and 32 age-matched controls were enrolled in our study. Each subject provided written informed consent, and this study was approved by the Institutional Review Board of the first affiliated hospital of Anhui medical university Subcommittee on Human Studies. Patients with SVCI met the previous criteria [17]. Exclusion criteria of SVCI including: a history of known stroke, head injury, Parkinson's disease, epilepsy, major depression or other neurological or psychiatric illness, severe visuo-spatial deficits, dentures or metallic stent *in vivo*. Twenty-three healthy controls were recruited from either the spouses of patients or recruited via advertisement. All subjects performed a neuropsychological battery assessment, including CAMCOG-C, MMSE, ADL and CDR Clinical dementia rating to evaluate the function of episodic memory, attention, psychomotor speed, executive function, visuo-spatial skills and emotion respectively.

### MRI data acquisition

Functional imaging data were acquired using a 3.0 T GE Signa HDxt MRI scanner (GE Milwaukee, WI, USA). At the beginning of the resting scan, the participants were asked to keep their eyes closed without falling asleep and relax. Resting state images were obtained using echo-planar imaging (EPI) sequence (TR=2 s, TE=30 ms, FOV=240 mm, flip angle=80°, matrix size=64 × 64, thickness=4 mm, gap=0.6 mm, number of slice=33 and time point=230). A 3D high-resolution T1-weighted anatomical images were also acquired using an inversion recovery fast spoiled gradient-recalled echo pulse sequence (TR=9.5 ms; TE=3.9 ms; TI=450 ms; flip angle=20°; field of view=256 mm; matrix size=512×512).

### FMRI data preprocessing

Functional connectivity analysis was performed on the MATLAB platform using the correlated and anticorrelated brain networks (CONN) Toolbox [18]. The main preprocessing procedure included discarding the first ten volumes, conventional slice timing correction, realignment, coregistration, normalization, and spatial smoothing with an 8-mm Gaussian kernel of full width at half-maximum. Head motion of more than 3 mm maximum displacement in any direction (x, y, and z) or 3 degrees of any angular motion throughout the course of the scan were excluded. We also compared the six head motion parameters. No significant difference was found in each parameter between the two groups (two sample *t* test, p>0.05). Moreover, to remove possible effects, six head motion parameters and the mean time series of gray matter, white matter and cerebrospinal fluid signals were introduced as covariates into the random effects model. A component-based noise correction method (Comp Cor) was also employed to reduce physiological and other noise artifacts [19]. A temporal band-pass filter (0.01< f <0.08 HZ) was performed to remove the effects of low-frequency drift and high-frequency noise.

### FMRI data analysis

**Figure 3:**
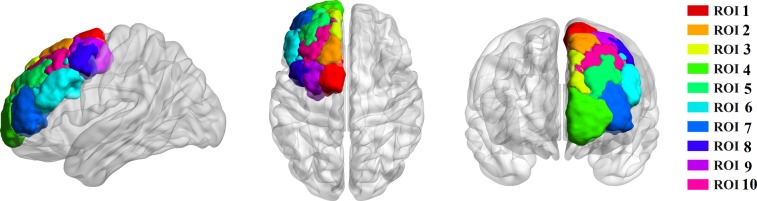
Tractography-based parcellation revealed ten clusters in human dorsal frontal cortex ROI 1 resembled SMA, ROI 2 resembled pre-SMA, ROI 3 resembled area9, ROI 4 resembled area10, ROI 5 resembled area 9/46d (e), ROI 6 resembled area 9/46v, ROI 7 resembled area 46, ROI 8 resembled area 8d, ROI 9 resembled rostral PMd, and ROI 10 resembled area 8v.

Ten subregions of the DFC in each hemisphere were defined in standard MNI space using the previous criteria, and then registered into each individual subject's MRI using FSL. We selected the DFC template as the ROI, which consists of 10 clusters (Figure 3). The results of the DFC parcellations in the form of atlases could be viewed in FSL (http://www.rbmars.dds.nl/CBPatlases.htm). It was noted that the DFC anatomical scheme appeared similar in both hemispheres according to a previous study [8].

Regional mean time series were obtained for each individual by averaging the fMRI time series over all voxels in each ROI. Pearson's correlation coefficients were calculated between the time courses of ROI and ROI, and a 10×10 symmetric correlation matrix were yielded, which contained normalized z-scores for each subjects. Results of exploratory analyses were considered significant if clusters survived family wise error (FWE) correction p<0.05.

### Voxel-based morphometry

To assess the influence of gray matter on functional connectivity, we analyzed our data for structural differences in ROIs between the groups using statistical parametric mapping (SPM) software and VBM toolbox. Structural MRI data processing was performed using VBM and the diffeomorphic anatomical registration using exponentiated Lie algebra (DARTEL) registration method, which has been reported in a previous study [20]. In addition, spatial smoothing with an 8-mm Gaussian kernel of full width at half-maximum was performed. Finally, the gray volume of 10 ROIs of DFC was calculated and the difference of ROI-wise gray volume between two groups was detected using two sample t test (p<0.05, false discovery rate correction).

### SVM-based classification

Connectivity matrices for each individual were converted to a feature vector containing 45 unique ROI-to-ROI connections (10×9/2).

**Figure 4:**
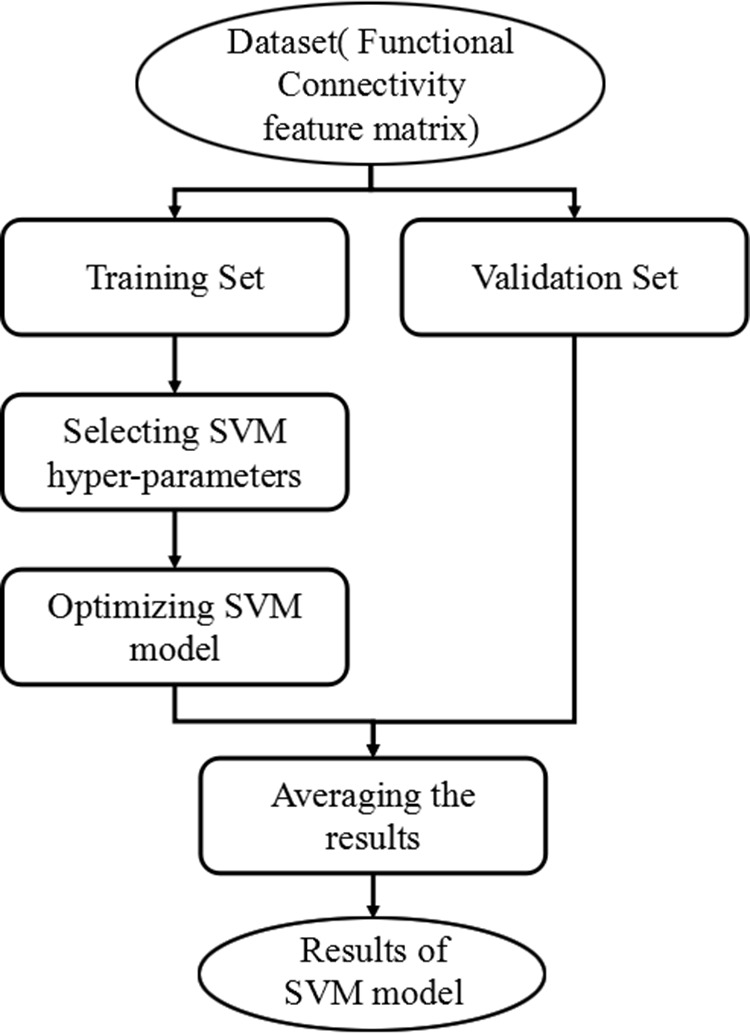
Flowchart of the proposed classification framework

SVM is one of the most powerful classification algorithms in terms of predictive accuracy. In this study, SVM-based classification method adopted radius bases function (RBF) as a kernel function because of its suitability for nonlinear mapping, few parameters and low numerical difficulty. A grid search algorithm was used to optimize the two parameters of SVM: γ, width of the RBF, and C, an input parameter for the SVM algorithm. The detailed description of the application of RBF kernel SVM in MRI data has been introduced in previous studies [21–22]. During the training phase, the SVM uses data was categorized into groups to determine the largest margin “hyperplane” to optimally separate the two groups. This process involves searching for a weight vector that maximizes the margin of separation between the groups by using the data points that are closest to the hyperplane as the defining points. These minimally distant data points are considered as “support vectors” and the classifier is thus fully specified by this subset of training samples. The flowchart of the proposed classification framework is shown in Figure 4.

To estimate the performance of the classifiers, 8-fold cross-validations were used. The performance of each classifier can be quantified using sensitivity (SS), specificity (SC) and generalization rate (GR) ROC curves were used to quantify the sensitivity and specificity of the classifier [23]. The AUC was used to quantitatively assess the classification power of a predictive model.

To assess the statistical significance of the observed classification accuracy, permutation tests were then employed to estimate the probability of obtaining GR higher than those obtained using the correct labels by chance [24]. We randomly assigned labels to each image and repeated the entire classification procedure 10,000 times and then counted the number of times that the GR for the permutated labels achieved higher than that obtained using the true labels.

### Correlation analysis

To identify the relationship between the strength of functional connectivity in DFC and the clinical scores in SVCI, the average strength of functional connectivity in the left and right DFC was extracted separately and correlated with the cognitive scores for all patients using Pearson's correlation analysis. Only the functional connectivity that differed significantly between groups was included in this analysis.

## CONCLUSIONS

This study used a MVPA method, which is based on functional connectivity pattern, to distinguish individuals with SVCI from the controls. The final model gave promising classification results with prediction accuracies from 45.38% to 75.07%. We proposed that functional connectivity within the DFC provided great potential for SVCI patient discrimination.
